# Heat transfer innovation of engine oil conveying SWCNTs-MWCNTs-TiO_2_ nanoparticles embedded in a porous stretching cylinder

**DOI:** 10.1038/s41598-024-65740-8

**Published:** 2024-07-16

**Authors:** Gunisetty Ramasekhar, A. Divya, Shaik Jakeer, S. R. R. Reddy, Ebrahem A. Algehyne, Muhammad Jawad, Ali Akgül, Murad Khan Hassani

**Affiliations:** 1 Department of Mathematics, Rajeev Gandhi Memorial College of Engineering and Technology (Autonomous), Nandyal, Andhra Pradesh 518501 India; 2 Department of Mathematics, Jesse College (Affiliated to North West Bangalore), Bengaluru, 560016 India; 3 Faculty of Mathematics, School of Technology, Apollo University, Chittoor, AP 517127 India; 4 Department of Mathematics, Koneru Lakshmaiah Education Foundation, Bowrampet, Hyderabad, 500043 India; 5 Department of Mathematics, Faculty of Science, University of Tabuk, P.O. Box 741, 71491 Tabuk, Saudi Arabia; 6 Department of Mathematics, The University of Faisalabad, Faisalabad, 38000 Pakistan; 7 Department of Computer Science and Mathematics, Lebanese American University, Beirut, Lebanon; 8 Department of Mathematics, Art and Science Faculty, Siirt University, 56100 Siirt, Turkey; 9 Department of Mathematics, Ghazni University, Ghazni, Afghanistan

**Keywords:** MHD, BVP Midrich scheme, Porous medium, Thermal radiation, Hybrid nanofluid, Engineering, Mathematics and computing, Nanoscience and technology

## Abstract

The influence of boundary layer flow of heat transfer analysis on hybrid nanofluid across an extended cylinder is the main focus of the current research. In addition, the impressions of magnetohydrodynamic, porous medium and thermal radiation are part of this investigation. Arrogate similarity variables are employed to transform the governing modelled partial differential equations into a couple of highly nonlinear ordinary differential equations. A numerical approach based on the BVP Midrich scheme in MAPLE solver is employed for solution of the set of resulting ordinary differential equations and obtained results are compared with existing literature. The effect of active important physical parameters like Magnetic Field, Porosity parameter, Eckert number, Prandtl number and thermal radiation parameters on dimensionless velocity and energy fields are employed via graphs and tables. The velocity profile decreased by about 65% when the magnetic field parameter values increases from 0.5 to 1.5. On the other hand increased by 70% on energy profile. The energy profile enhanced by about 62% when the Radiation parameter values increases from 1.0 < *Rd* < 3.0. The current model may be applicable in real life practical implications of employing Engine oil-SWCNTs-MWCNTs-TiO_2_ nanofluids on cylinders encompass enhanced heat transfer efficiency, and extended component lifespan, energy savings, and environmental benefits. This kind of theoretical analysis may be used in daily life applications, such as engineering and automobile industries.

## Introduction

In particular, sun-oriented collectors have been the focus of researcher’s interest as they investigate the possibility of using tiny fluids to improve heat transfer. The use of ordinary fluids such as ethylene glycol, drinking water, candle oil, and other similar substances is not sufficient to improve heat transfer. Researchers have carried out a great number of studies with the intention of enhancing the pace at which heat is transferred or transferred. Following this, they came to the conclusion that breakdown and blockage are the key factors responsible for the decrease in rising mass and heat transfer rates observed. Nanofluids are introduced to lessen such problems. In fact, Choi^1^ used the term "nanofluids" to refer to fluids containing dispersed tiny particles, which are most probably particles with sizes between one and one hundred nanometers. In some base fluids these nanoparticles are suspended. The main challenge facing the younger researchers in this sector is the development of better heat transfer techniques. Most upgrading processes fall into one of two categories. This relates to certain latent and dynamic tactics. Specialized latent processes need special computations, liquid additives, heated pressing, and so on. Dynamic techniques that include magnetic and electric fields need external powers. To improve the thermal performance of common fluids, including oil, water, and ethylene–glycol combinations, liquid additive chemicals are used most upgrading processes fall into one of two categories. This relates to certain latent and dynamic tactics. Specialized latent processes need special computations, liquid additives, heated pressing, and so on. Dynamic techniques that include magnetic and electric fields need external powers. To improve the heat transfer excellence of common fluids, including oil, water combinations, liquid additive chemicals are used Hayat et al.^2^. The impact of a strong fluid combination on essential heat transfer enhancement has been extensively studied by many investigators. It has been noted that a number of factors, including clogging, abrasion, extra pressure loss, etc., contributed to the mixture's incorrect use in upgrading thermal execution. Nanofluids have solved all of these problems and they also offer some very notable advantages, such as strong heat conductivity at lower nanoparticles concentrations, little halting in stream sections, homogeneity and long-term strength. The unique qualities that these materials possess increase their value across a wide range in industries and biomedicine fields. The unique sliding properties of Brownian motion and the creation of thermophoresis around the nanofluid were studied by Buongiorno^3^. Gupta et al.^4^ conducted research on radiative MHD nanofluid across an inclined, stretched cylinder. So many authors did the similar research analysis they are Yaswin et al., Hayat et al., Salahuddin et al., Chinnaswamy et al., and Saha et al.^5–9^.

For all intents and purposes, tiny fluids are preferable than traditional liquid because of improving the amount of heat that is transmitted. A stimulus is applied to the composite nanofluid in order to optimize its performance. The only two distinct kinds of tiny particles that are present in a fluid are those that make up a hybrid nanofluid. At the present moment, it is currently demonstrated that nanofluids that are hybrid possess a higher capacity for heat transfer compared to either ordinary tiny fluids or different operating liquids Acharya et al.^10^. Because these hybrid nanofluids are superior than conventional nanofluids in terms of their ability to transport heat, they find applications in a wide variety of industries, including electrical cooling, automotive heat administration, welding process, electrical systems, greasing, hydropower fabrication, newspaper and biomedical creation, atomic section, rocket component manufacture, and a number of others Divya and Reddy^11^. By considering nanoparticles that are mixed in the regular liquids and formed as hybrid nanofluids mixed with hyperbolic tangent fluid and this mathematical model for analyzing heat transfer has been illustrated by Alharbi, Nawaz, and Nazir^12^. When comparing the thermal characteristics of the mixed tiny fluids, the finite element method is employed to determine the answer for the computational models that have been developed. In the case of a hybrid tiny fluids, the motion of the stagnation point is directed into a cylindrical structure that is both expanding and swelling is investigated by Waini, Ishak and Pop^13^. In the context of investigating the thermal and mass transfer in hybrid nanoliquids flow over a stretching cylinder by incorporating a magnetic dipole, a comparative study on the flow of two different combinations of hybrid nanofluids was illustrated by Kumar et al.^14^. So many authors investigated by Alsaedi, Muhammad and Hayat, Reddisekhar et al., Hayat and Nadeem, Karthik et al., Jayadevamurthy et al.^15–19^.

In the past few years, porous media has gained importance has received the attention it deserves in more and more important domains. A porous medium increases the area of contact between a fluid and a strong surface, increasing the scattering of nanoparticles and leading to an enhanced in the achievement proportion. Consequently, the importance of commonplace heat works has enlarged Shaw et al., Ramasekhar and Bala Anki Reddy^20,21^. Al-Farhany and Abdulsahib^22^ conducted a numerical analysis in porous media to examine heat transfer of mixed convective phase, which is divided into two layers in a square enclosure with in a spinning circular cylinder at the center of the cavity. In a porous medium with magneto-tiny nanofluid flow along with the heat generation effects has been explored by Eid and Nafe, Waini et al., Pavithra and Gireesha, Mebarek-Oudina et al., Jangid et al., Jangid et al., Jangid et al., Hussain, Syed et al., Varun Kumar et al., Madhukesh et al., Jyothi et al., Wang et al., Wang et al., Wang et al.^23–36^.

Therefore, inspired from the above literature, the current study theoretically examined by the simultaneous effects of magnetohydrodynamic, porous medium, thermal radiation hybrid nanofluid flow over a stretching cylinder and which all are taken into the present model. As per the author knowledge this kind of model has not been examined before. The mathematical equations are created in the form of partial differential equations. Using appropriate self-similarity variables, we can transform the partial differential equations into ordinary differential equations. After applying transformations, for graphical purpose we have used the numerical method that is BVP Midrich scheme in MAPLE solver. In the results and discussion section, graphs for different physical significance are given. Hybrid nanofluid with carbon nanotubes, titanium dioxide, and engine oil enhances for solar thermal efficiency. Applications include concentrated solar power, thermal collectors, and lubrication in solar tracking. As a consequence, the researchers are certain that the latest research is unique, will have a significant impact in the fields of technology and mathematics, and has the potential to inspire young scientists.

## Research questions

- Does the heat transfer rate enhance due to the adoption of hybrid SWCNTs-TiO_2_ nanoparticles along with Engine oil nanofluid than MWCNTs-TiO_2_ nanofluid?
- How magnetic field influences the fluid velocity?
- Does the increasing value of curvature parameter (cylinder) and Eckert number delay the boundary layer separation and reduce the heat transfer rate?
- What are the industry and automobile implications of incorporating hybrid nanofluids in the magnetohydrodynamic flow dynamics over a cylinder?
- What numerical methods and simulations are mainly successful for investigating the magnetohydrodynamic behavior of hybrid Engine oil-based nanofluid flow over a cylinder in automobile applications?
- How does the addition of hybrid Engine oil-based nanofluid with impact of the magnetohydrodynamics behavior in the context of flow over a porous cylinder?

### Feature scope

This investigation can be expanded to include different nanoparticle categories, temperature boundary conditions, particle shapes, various types of non-Newtonian fluids and transient movement consequences. This is additionally adaptable to different numerical methods, making it applicable to a wide range of technological as well as scientific uses (Table 1).

**Table 1:** Thermophysical properties of base fluid and hybrid nanofluids Ahmad et al., and Farooq et al.^41,42^.

Property	Engine oil	SWCNTs	MWCNTs	TiO_2_
Density *ρ* (kg m^−3^)	884.00	2600.00	1600.00	4250
Specific heat *C*_*p*_ (J kg^−1^ K^−1^)	1910.00	425.00	796.00	686.2
Heat conductivity *k*_*f*_ (W m^−1^ K^−1^)	0.1440	6600.00	3000.0	8.9538
Electrical conductivity *σ* (Ω m^−1^)	10^−6^–2 × 10^−9^	10^−16^–10^8^	10^6^–10^7^	10^−12^
Pr	6450			

## Mathematical modeling

- A steady two-dimensional with the combination of SWCNTs-MWCNTs-TiO_2_ nanoparticles along with Engine oil as a base fluid analyzed by stretching cylinder.
- The cylinder contains with a radius “*a*”.
- In the presence of magnetohydrodynamic is examined.
- In this model *u* and *v* are velocity components.
- Both the porous medium and the thermal radiation are being investigated for the purpose of motivation.
- It is important for conducting a comparative investigation for two distinct cases like Case (i) (SWCNTs-TiO_2_/EO) and Case (ii) (MWCNTs-TiO_2_/EO).

**Figure 1:**
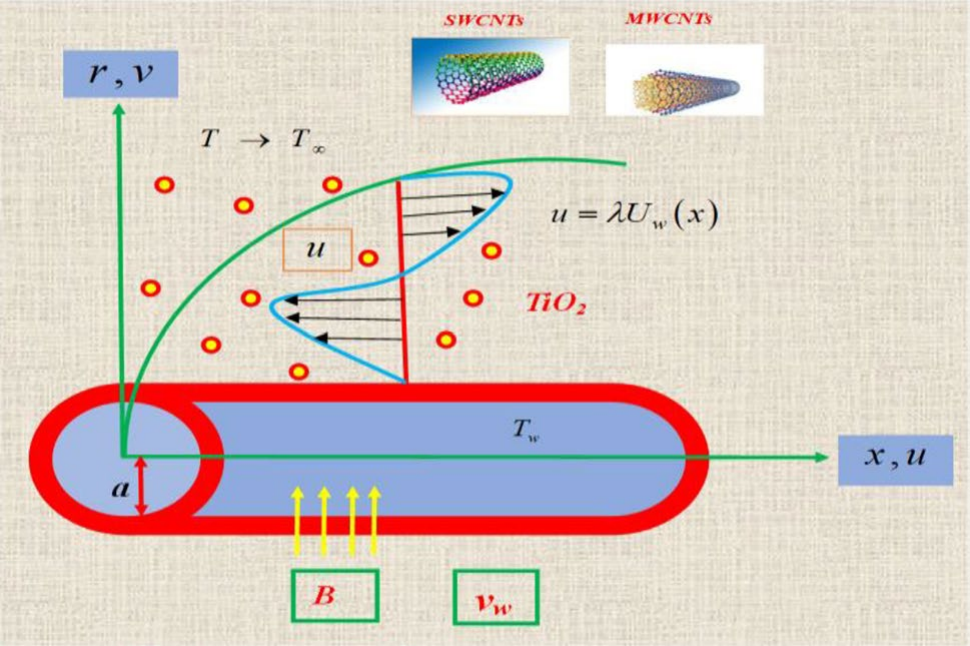
Flow justification of the model.

By considering the assumptions of the fluid flow governing equations are defined (Fig. 1).

The mathematical flow equations are constructed as Najiyah et al., Awan et al., Umeshaiah et al., Rashad et al.^37–40^:

1 $$\frac{\partial u}{{\partial x}} + \frac{\partial v}{{\partial r}} = 0,$$

2 $$u\frac{\partial u}{{\partial x}} + v\frac{\partial u}{{\partial r}} = \frac{{\mu_{hnf} }}{{\rho_{hnf} }}\left( {\frac{{\partial^{2} u}}{{\partial r^{2} }}} \right) - \frac{{\mu_{hnf} }}{{\rho_{hnf} }}\frac{u}{{K^{ * } }} - \frac{{\sigma_{hnf} }}{{\rho_{hnf} }}\left( {B^{2} u} \right),$$

3 $$u\frac{\partial T}{{\partial x}} + v\frac{\partial T}{{\partial r}} = \frac{{k_{hnf} }}{{\left( {\rho c_{p} } \right)_{hnf} }}\left( {\frac{{\partial^{2} T}}{{\partial r^{2} }}} \right) + \frac{1}{{\left( {\rho c_{p} } \right)_{hnf} }}\frac{\partial }{\partial r}\left( {\frac{{16\sigma^{*} T_{\infty }^{3} }}{{3kk^{*} }}\frac{\partial T}{{\partial r}}} \right) + \frac{{\sigma_{hnf} }}{{\left( {\rho c_{p} } \right)_{hnf} }}B^{2} u^{2} .$$

Subjected to Najiyah et al.^37^

4 $$\begin{gathered} v = v_{w} \left( r \right),\quad u = \lambda U_{w} \left( x \right),\quad T = T_{w} \left( x \right),\quad at\quad r = a \hfill \\ u \to 0,\quad T \to T_{\infty } ,\quad \quad as\quad r \to \infty . \hfill \\ \end{gathered}$$

The following suitable self-similarity transformations are defined as Najiyah et al.^37^:

5 $$u = \frac{{u_{0} x}}{L}f^{{\prime }} \left( \eta \right),\quad v = - \frac{a}{r}\sqrt {\frac{{u_{o} \upsilon_{f} }}{L}} f\left( \eta \right),\quad \theta \left( \eta \right) = \frac{{T - T_{\infty } }}{{T_{w} - T_{\infty } }},\quad \eta = \sqrt {\frac{{u_{o} }}{{\upsilon_{f} L}}} \frac{{r^{2} - a^{2} }}{2a},$$

So that

6 $$v_{w} \left( r \right) = - \frac{a}{r}\sqrt {\frac{{u_{o} \upsilon_{f} }}{L}} S.$$

Thermophysical properties of *hnf* are

$$W_{1} = \frac{{\mu_{hnf} }}{{\mu_{f} }},\quad W_{2} = \frac{{\rho_{hnf} }}{{\rho_{f} }},\quad W_{3} = \frac{{(\rho c_{p} )_{hnf} }}{{(\rho c_{p} )_{f} }},\quad W_{4} = \frac{{k_{hnf} }}{{k_{f} }},\quad W_{5} = \frac{{\sigma_{hnf} }}{{\sigma_{f} }}.$$

7 $$\left. \begin{gathered} W_{1} = \frac{1}{{\left( {1 - \phi_{1} } \right)^{2.5} \left( {1 - \phi_{2} } \right)^{2.5} }}, \hfill \\ W_{2} = \left\{ {\left( {1 - \phi_{2} } \right)\left[ {\left( {1 - \phi_{1} } \right) + \phi_{1} \left( {\frac{{\rho_{{s_{1} }} }}{{\rho_{f} }}} \right)} \right] + \phi_{2} \frac{{\rho_{{s_{2} }} }}{{\rho_{f} }}} \right\}, \hfill \\ W_{3} = \left( {1 - \phi_{2} } \right)\left[ {\left( {1 - \phi_{1} } \right) + \phi_{1} \left( {\frac{{(\rho c_{p} )_{{s_{1} }} }}{{(\rho c_{p} )_{f} }}} \right)} \right] + \phi_{2} \frac{{(\rho c_{p} )_{{s_{2} }} }}{{(\rho c_{p} )_{f} }}, \hfill \\ W_{4} = \frac{{k_{{s_{1} }} + 2k_{bf} - 2\phi_{2} (k_{bf} - k_{{s_{2} }} )}}{{k_{{s_{2} }} + 2k_{bf} + \phi_{2} (k_{bf} - k_{{s_{2} }} )}} \times \frac{{k_{{s_{1} }} + 2k_{f} - 2\phi_{1} (k_{f} - k_{{s_{1} }} )}}{{k_{{s_{1} }} + 2k_{f} + \phi_{1} (k_{f} - k_{{s_{1} }} )}}, \hfill \\ W_{5} = \frac{{\sigma_{{s_{2} }} + 2\sigma_{nf} - 2\phi_{2} (\sigma_{nf} - \sigma_{{s_{2} }} )}}{{\sigma_{{s_{2} }} + 2\sigma_{nf} + \phi_{2} (\sigma_{nf} - \sigma_{{s_{2} }} )}} \times \frac{{\sigma_{{s_{1} }} + 2\sigma_{f} - 2\phi_{1} (\sigma_{f} - \sigma_{{s_{1} }} )}}{{\sigma_{{s_{1} }} + 2\sigma_{f} + \phi_{1} (\sigma_{f} - \sigma_{{s_{1} }} )}}. \hfill \\ \end{gathered} \right\}$$

The transformation in Eq. (5) obeys the continuity Eq. (1), hence, Eqs. (2)–(4) are converted into following system of ordinary differential equations:

8 $$\frac{{W_{1} }}{{W_{2} }}\left( {\left( {1 + 2\gamma \eta } \right)f^{\prime \prime \prime } + 2\gamma f^{\prime \prime } } \right) + ff^{\prime \prime } - \left( {f^{\prime } } \right)^{2} - \frac{{W_{1} }}{{W_{2} }}Kf^{\prime } - \frac{{W_{5} }}{{W_{2} }}Mf^{\prime } = 0,$$

9 $$\frac{1}{\Pr }\left( {\left( {\frac{{W_{4} }}{{W_{3} }}\left( {1 + 2\gamma \eta } \right)\left( {1 + Rd} \right)\theta^{\prime \prime } + 2\gamma \theta^{\prime } )} \right) + \left( {\frac{1}{{W_{3} }}Rd\gamma \theta^{\prime } } \right)} \right) + f\theta^{\prime } - 2f^{\prime } \theta + \frac{{W_{5} }}{{W_{3} }}EcM\left( {f^{\prime } } \right)^{2} = 0.$$

Transformed boundary conditions are

10 $$\begin{gathered} f\left( 0 \right) = S,\quad f^{\prime } \left( 0 \right) = \lambda ,\quad \theta \left( 0 \right) = 1 \hfill \\ f^{\prime } \left( \infty \right) = 0,\quad \theta^{\prime } \left( \infty \right) = 0. \hfill \\ \end{gathered}$$

**Table:** 

$M = \frac{{\sigma_{f} B^{2} L}}{{\rho_{f} u_{0} }}$ Magnetic field parameter	$\gamma = \sqrt {\frac{{\upsilon_{f} L}}{{u_{o} a^{2} }}}$ Curvature parameter
$Pr = \frac{{\mu_{f} \left( {c_{p} } \right)_{f} }}{{k_{f} }}$ is the Prandtl number	$K = \frac{{\upsilon_{f} }}{{U_{w} K^{ * } }}$ is the porosity parameter
$Rd = \frac{{16\sigma^{*} T_{\infty }^{3} }}{{3k^{*} k_{f} }}$ is the thermal radiation parameter	$Ec = \frac{{U_{w}^{2} }}{{c_{p} \left( {T_{w} - T_{\infty } } \right)}}$ is the Eckert number

The dimensional form of *C*_*f*_ and *Nu*_*x*_ are given by

11 $$\begin{aligned} C_{f} & = \frac{{\mu_{hnf} }}{{\rho_{f} U_{w}^{2} }}\left( {\frac{\partial u}{{\partial r}}} \right)_{r = 0} , \\ Nu_{x} & = \frac{{xk_{hnf} }}{{k_{f} \left( {T_{w} - T_{\infty } } \right)}}\left( { - \frac{\partial T}{{\partial r}}} \right)_{r = a} . \\ \end{aligned}$$

The non-dimensional form of Eq. (11) converted is

12 $$Re_{x}^{{{\raise0.7ex\hbox{$1$} \!\mathord{\left/ {\vphantom {1 2}}\right.\kern-0pt} \!\lower0.7ex\hbox{$2$}}}} C_{f} = Cf^{*} = W_{1} f^{{{\prime \prime }}} \left( 0 \right),\quad Re_{x}^{{ - {\raise0.7ex\hbox{$1$} \!\mathord{\left/ {\vphantom {1 2}}\right.\kern-0pt} \!\lower0.7ex\hbox{$2$}}}} Nu_{x} = Nu^{*} = - W_{4} \theta^{{\prime }} \left( 0 \right).$$

where $Re_{x} = {{U_{w} x} \mathord{\left/ {\vphantom {{U_{w} x} {v_{f} }}} \right. \kern-0pt} {v_{f} }}$ is the local Reynolds number.

## Solution methodology

**Figure 2:**
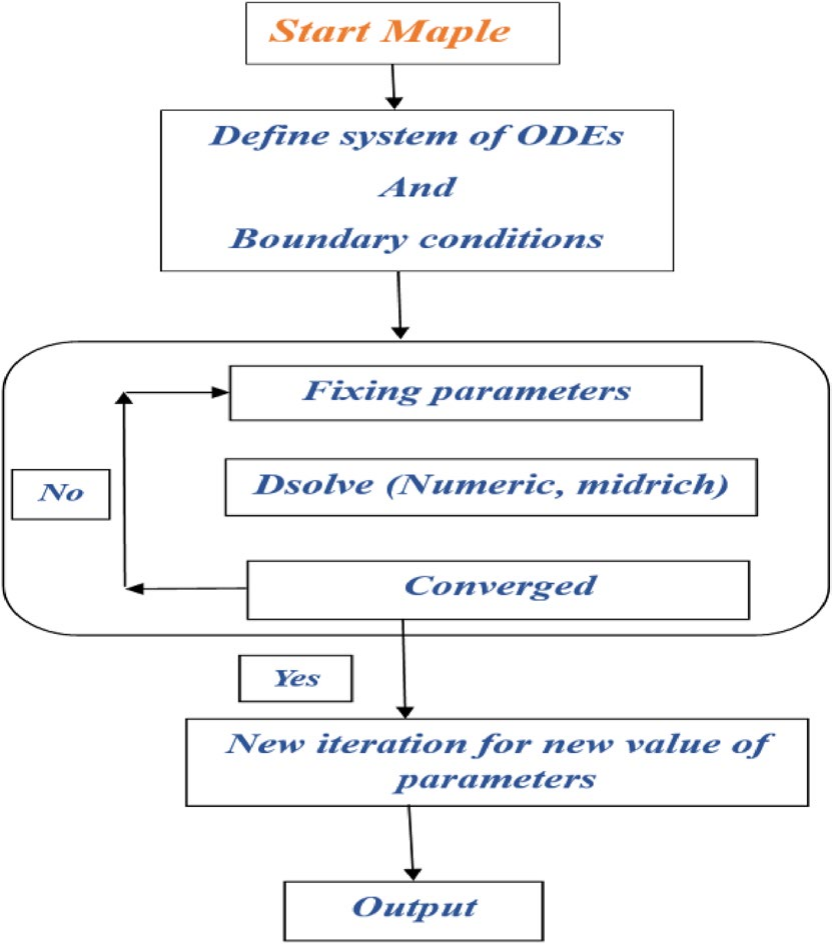
A flow chart pictogram of BVP Midrich technique.

The nature of the ODE system (8–9) with BCs (10) is extremely nonlinear in its characteristics. For the purpose of dealing with these equations, we adopt a computational approach known as the BVP Midrich method. Using Maple, we are able to solve the control problem. The midway method's standard operating procedure is laid out in detail below (Fig. 2) Ramasekhar^42^.

The following is a demonstration of the overall algorithm for the technique of midpoint collocation

13 $$\overline{{Z^{\prime } }} \left( x \right) = F\left( {x,\overline{Z} (x)} \right),\quad \overline{Z} (x_{0} ) = \overline{{Z_{0} }}$$

In the explicit midpoint approach, also known as the Modified Euler method, the formula looks like this

14 $$\overline{{Z_{n + 1} }} = \overline{{Z_{n} }} + hF\left( {x_{n} + \frac{h}{2},\overline{{Z_{n} }} + \frac{h}{2}F\left( {x_{n} ,\overline{{Z_{n} }} } \right)} \right)$$

The above equation *h* represents the step size and $x_{n} = (x_{0} + nh)$.

The strategy that takes into account the implicit midpoint may be described as

15 $$\overline{{Z_{n + 1} }} = \overline{{Z_{n} }} + hF\left( {x_{n} + \frac{h}{2},\overline{{Z_{n} }} + \frac{1}{2}\left( {\overline{{Z_{n} }} ,\overline{{Z_{n + 1} }} } \right)} \right),\quad n = 0,1,2, \ldots$$

At each step size, the technique for locating the midpoint has a local error of order *O*(*h*^3^) whereas the global error is of order *O*(*h*^2^). When dealing with algorithms that are more quantifiable demanding, the algorithm-error decreases at a quicker rate as $h \to 0$ progresses, and the result gets more dependable Ramasekhar et al.^43^.

## Results and discussion

This section provides the results of figures that demonstrate the influence of numerous important factors are magnetic field, Eckert number, porosity parameter, curvature parameter and thermal radiation along With their ranges, $0.5 < M < 1.5,\;\;0.3 < K < 0.9,\;\;0.1 < \gamma < 0.3$, $0.2 < M < 0.6,\;\;0.5 < Ec < 1.5,\;\;1.0 < Rd < 3.0$ we evaluate the parameter values and also we considered Pr value of the Engine oil is 6450 along with two different cases of hybrid nanofluids are considered in the present manuscript. When compared to nanofluid hybrid nanofluid to generate better performance. During this phase of the research, hybrid rheological model’s behavior was outlined using the BVP Midrich scheme. They are contrasted in both SWCNTs and MWCNTs fluid flows with the combination of TiO_2_, suspended in the regular fluid called engine oil scenarios. With the significant exception of those values which is used for Table 2 comparisons, whose numerical values are fixed. The general values of the parameters are considered as $\phi_{1} = \phi_{2} = 0.05,\gamma = K = M = 0.1,Rd = 0.5$, $Ec = 0.2,\;S = \lambda = 1.0,\;\Pr = 6450$.

**Table 2:** The quantitative results of the *Cf* of different of values for ϕ_2_ by fixing parameter values *S* = *γ* = *M* = *Ec* = 0, and *Pr* = 6.135, ϕ_1_ = 0.1,* λ* = 1.

ϕ_2_	Najiyah et al.^37^	Present results
0.005	− 1.327098	− 1.327087
0.02	− 1.409490	− 1.409496
0.04	− 1.520721	− 1.520718
0.06	− 1.634119	− 1.634127

### Velocity profiles

**Figure 3:**
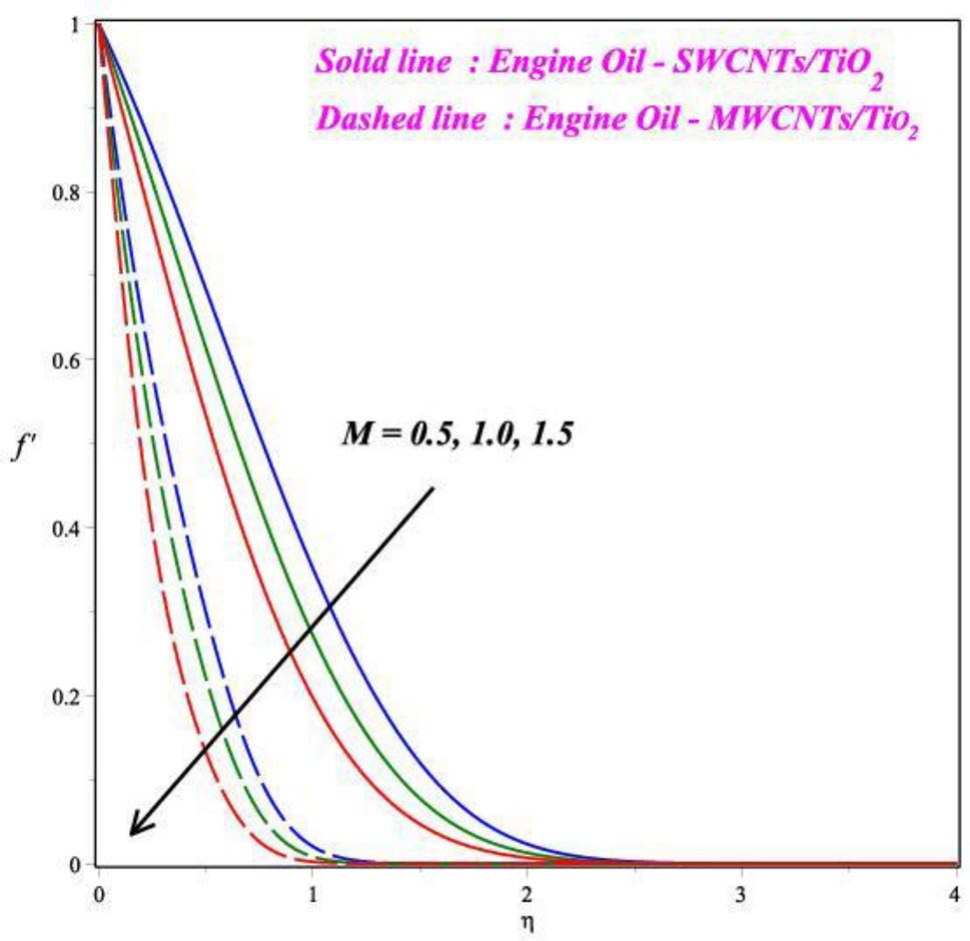
Influence of *M* on *f*′(η).

**Figure 4:**
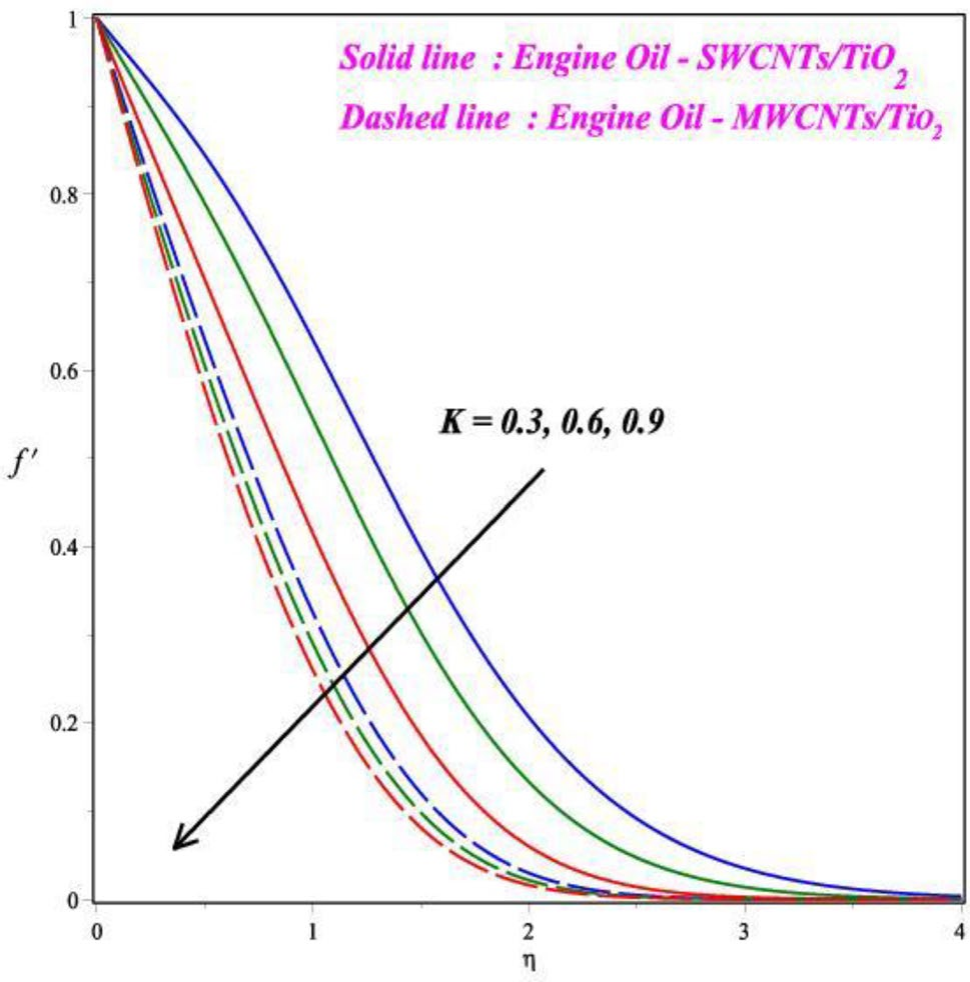
Impact of *K* on *f*′(η).

**Figure 5:**
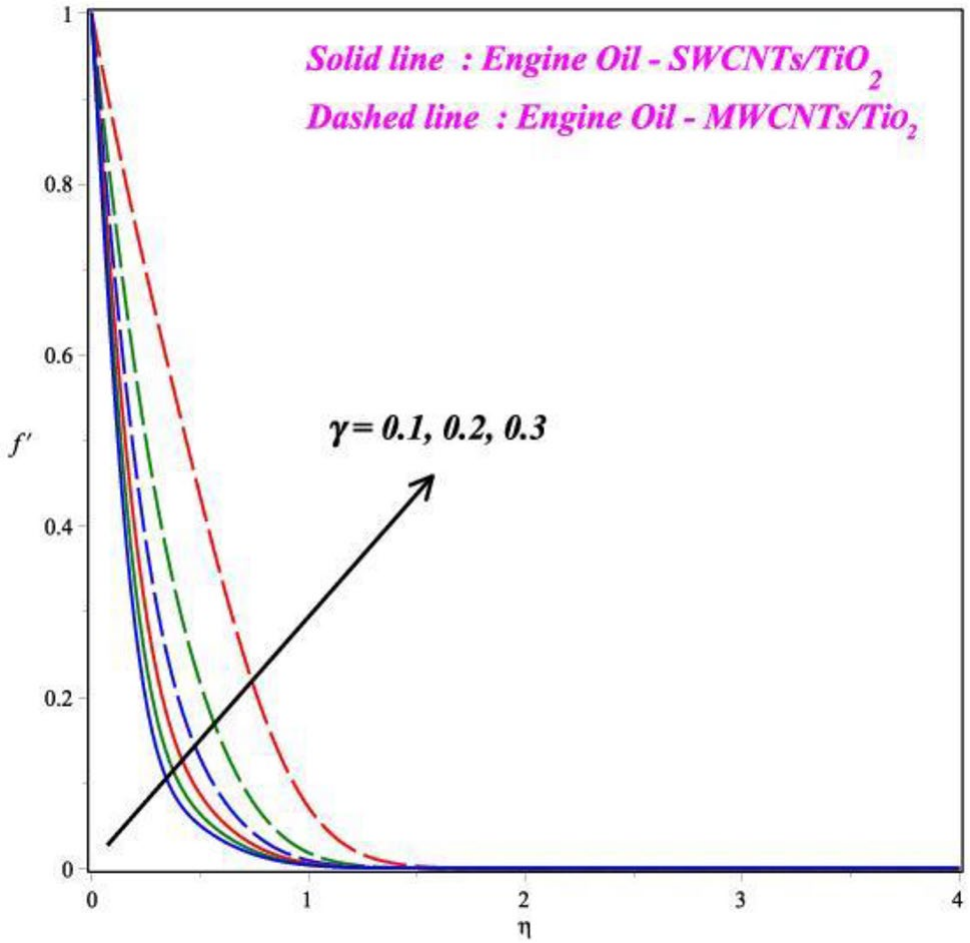
Impact of *γ* on *f*′(η).

Figures 3, 4 and 5 illustrate the pictorial representation of the fluid velocity dispersion, which may also be used for acquiring new numerical outcomes. As the *M* values rise, the *f*′(*η*) outline decreased, as Fig. 3 illustrates. For increasing values of magnetic field parameter values decreased the velocity profile. The velocity profile decreased by about 65% when the *M* parameter values increases from 0.5 to 1.5. In practical terms, more grounded Lorentz powers are produced by larger magnetic field parameter values, and this eventually lowers the velocity profile. Being aware that the velocity of the fluid through this medium is inversely related to the viscosity, leading to a phase when the velocity of the nanofluid decreases. The detrimental effect of the *K* on the *f*′(*η*) is apparent from Fig. 4 in both comparative cases. It is shown that the velocity outline declines for case ii with increasing *K* parameter, which is in tune with reality. For increasing values of porosity parameter values decreased the velocity profile. The velocity profile decreased by about 90% when the *M* parameter values increases from 0.3 to 0.9. Furthermore, when we the trip away from the border, as far the liquid movement is concerned, the porous nature of the border is insignificant. As shown in Fig. 5, the curvature parameter *γ* has an impact on fluid speed. The Comparison between two hybrid nanofluid cases the effect of curvature parameter is seen in Fig. 5. For the higher values of curvature parameter values increased the velocity profile. The velocity profile increased by about 50% when the curvature parameter values increases from 0.1 to 0.3. The hybrid fluid's velocity rises near the surface and decreases away from the boundary as the curvature parameter's value increases. In hypothesis, there is a reverse correlation between the cylinder's radius and curvature. Consequently, as the *γ* enhances, the cylinder's radius reduced. Due to this, there was less fluid connection along the surface of the cylinder. Therefore, the fluid particles cause very little friction on the surface. Hence, the fluid velocity asymptotically increases with a large increase in the curvature parameter *γ*.

### Energy profiles

**Figure 6:**
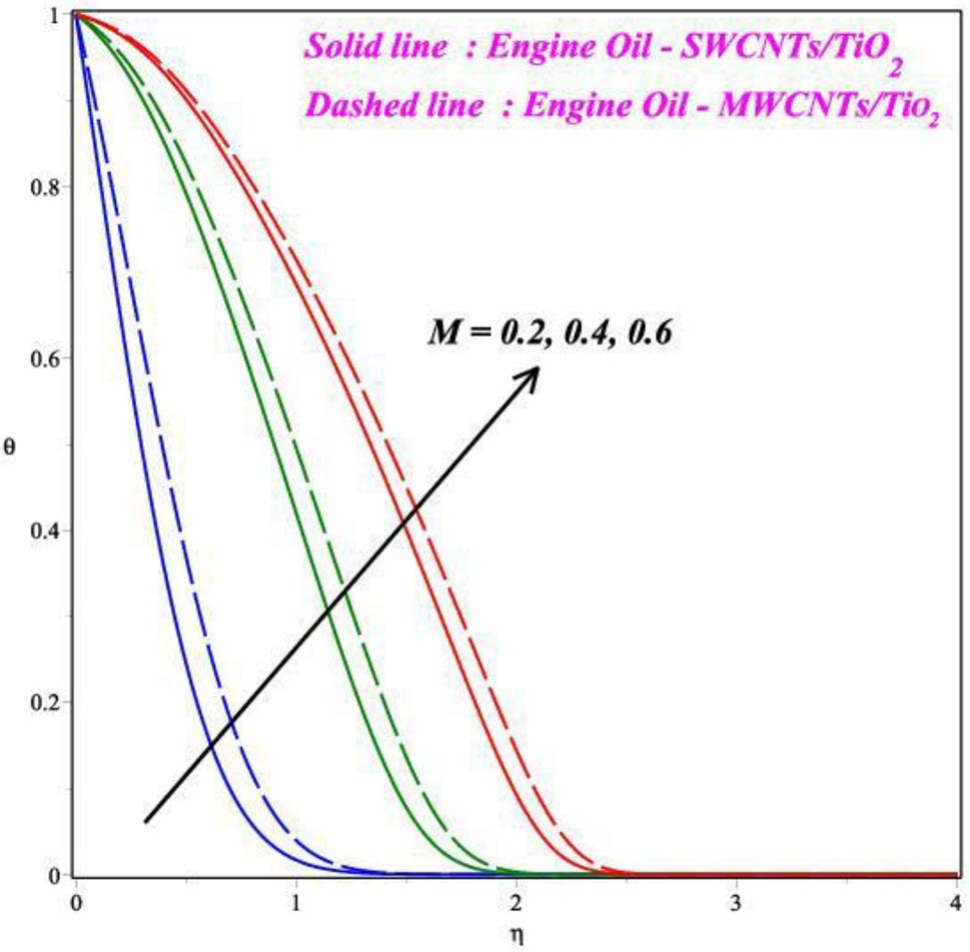
Impact of *M* on *θ*(*η*).

**Figure 7:**
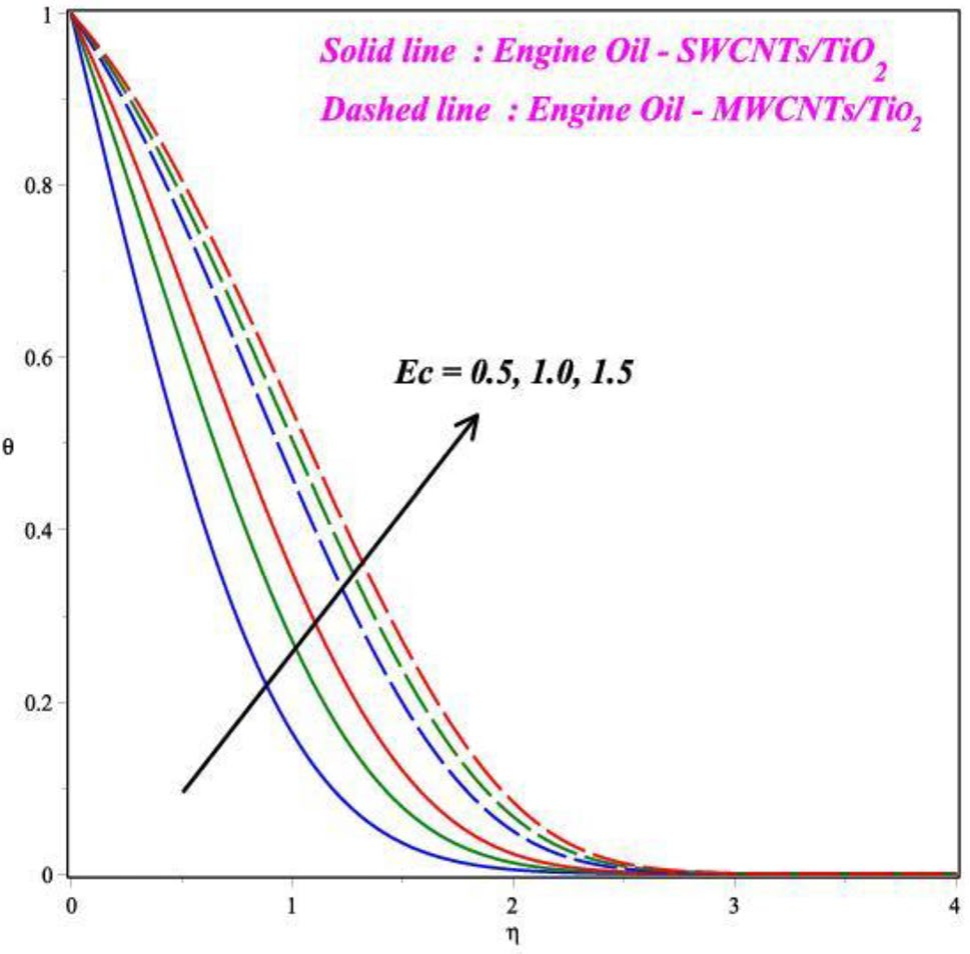
Impact of *Ec* on *θ*(*η*).

**Figure 8:**
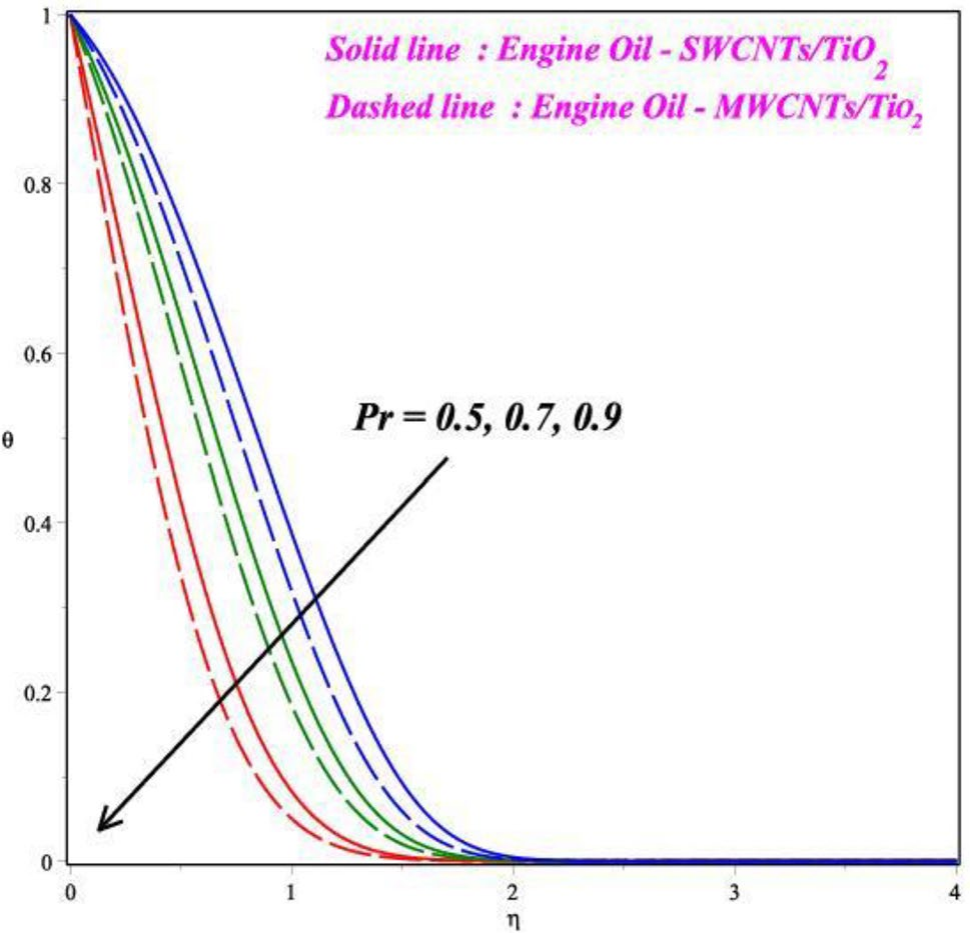
Impact of *Pr* on *θ*(*η*).

**Figure 9:**
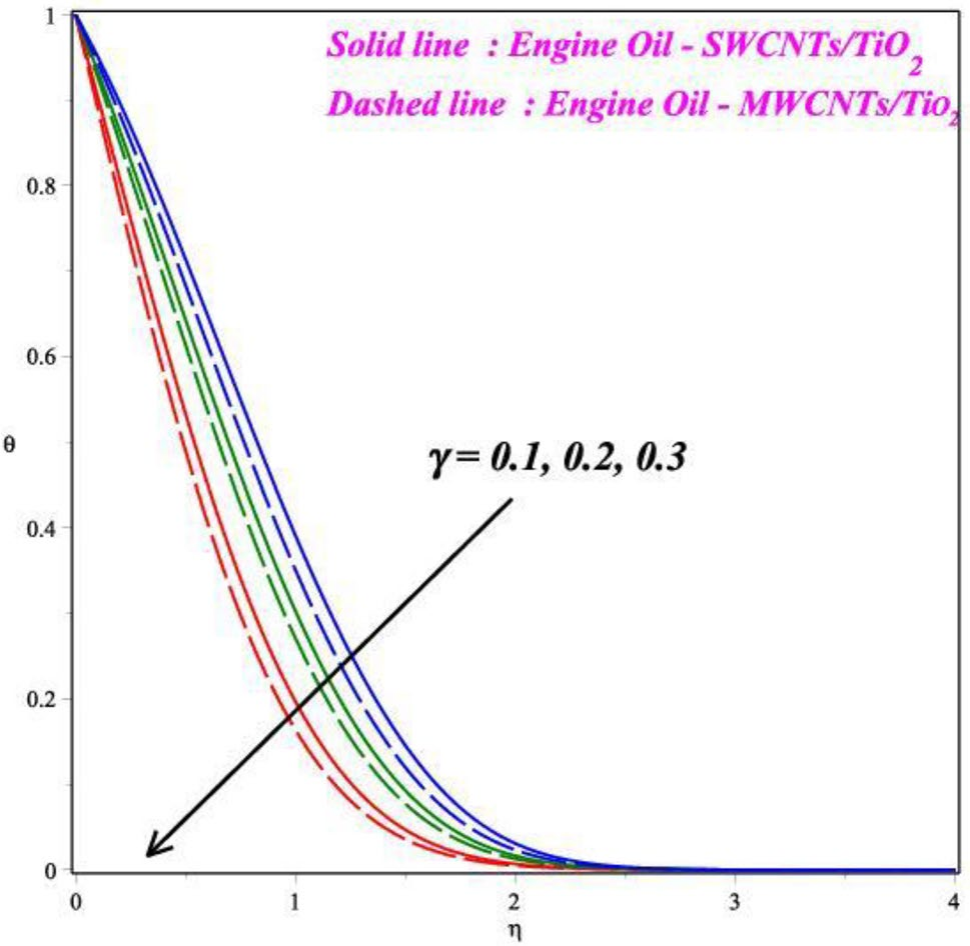
Impact of *γ* on *θ*(*η*).

**Figure 10:**
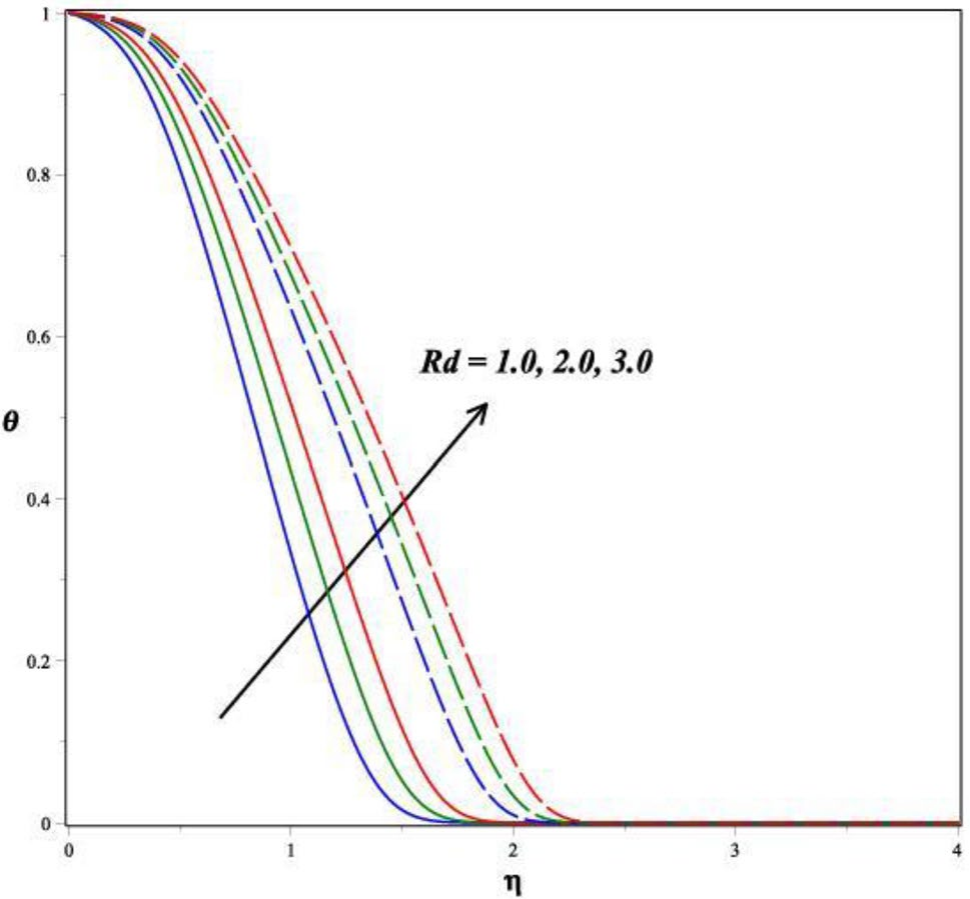
Impact of *Rd* on *θ*(*η*).

Figures 6, 7, 8 and 9 demonstrates the vital effects of *M*, *Ec*, Pr and *γ* on the temperature profile *θ*(*η*) of the two different cases. As the *M* increases, the energy *θ*(*η*) profile fluctuations grow, which is shown in Fig. 6. For increasing values of magnetic field parameter values enhanced the energy profile. The energy profile increased by about 70% when the *M* parameter values increases from 0.2 to 0.6. The force of Lorentz, which connects resistance to fluid temperature, enhances with an enlarge in the *M* parameter, necessitating temperature consequences. In another perspective, more electron collisions occur when the resistive force is higher, increasing the heat produced in the fluid flow. As the *M* increases, the energy profile increased. Expanding outcomes regarding greater estimates of the temperature profile's *θ*(*η*) Eckert number *Ec* are presented in Fig. 7. Actually, because of the frictional heating, more dynamic thermal energy is deposited in the fluid particles. For increasing values of Eckert number values enhanced the energy profile. The energy profile increased by about 75% when the *Ec* parameter values increases from 0.5 to 1.5. As a result, the temperature field supports estimates of increasing Eckert numbers in both SWCNT’s and MWCNT’s fluid flows with the combination of TiO_2_, suspended in the base fluid scenarios. Figure 8 exhibits the consequences of a hybrid nano liquid with a Pr on the energy *θ*(*η*). As a result, the impacts of the Prandtl number Pr on the fluid temperature profile are acknowledged. In this hybrid model, the Prandtl number increases to indicate moderate augmentations in the liquid temperature. In accordance with the theory, the Prandtl number and thermal diffusivity are negatively correlated. Thermal diffusivity erodes, resulting in a decrease in thermal layer thickness as the Prandtl number increases. The result is a reducing direction of thermal field intensity. Figure 9 shows that how the *γ* affects the hybrid fluid's energy profile *θ*(*η*). As the *γ* is increased, the heat transfer rate decreases. The upshot of this is that the dimension of the thermal barrier layer decreases, which in turn causes an increase in energy. The stretching cylinder has a predominant temperature because it is more accommodating to the passage of liquid. Figure 10 shows how the *Rd* affects the hybrid fluid's temperature profile *θ*(*η*). The energy profile increased by about 62% when the *Rd* parameter values increases from 1.0 to 3.0. As *Rd* parameter is enlarged, the heat transmission rate enhanced, resulting in an increase in the dimension of the thermal barrier layer. Physically, As Rd is increased, a greater amount of heat is transferred into the liquid, which results in the thermal barrier becoming strengthened.

### Cf* and Nu* profiles

**Figure 11:**
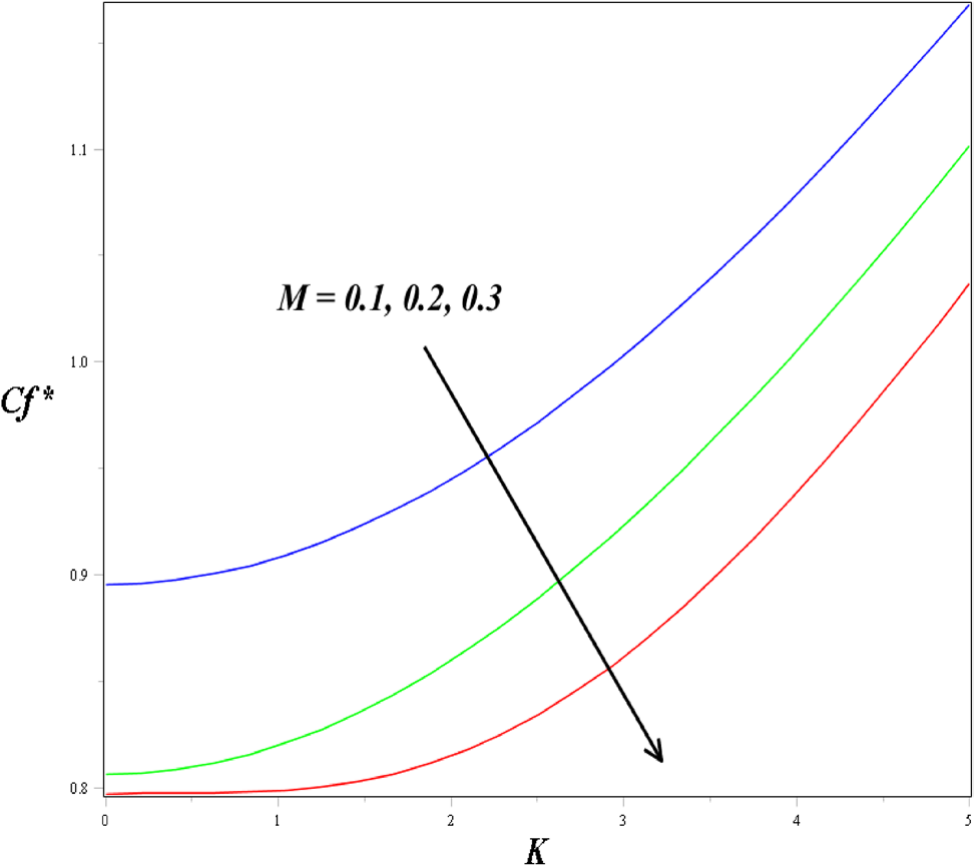
Impact of *M* and *K* on Cf^*^.

**Figure 12:**
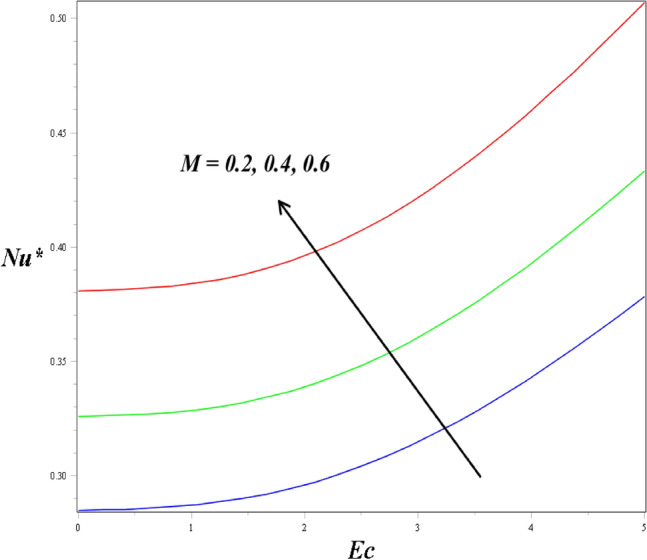
Impact of *M* and *Ec* on Nu^*^.

The associations between the Cf* and Nu* by the porosity parameter, Eckert number and magnetic field are highlighted in Figs. 11 and 12. Figure 11 shows that the impacts of porosity and the magnetic field parameter on the Cf*, for the higher values of the magnetic field parameter the skin friction coefficient profile enhanced. The impact of the magnetic field and the Eckert number also contribute to increasing the rate of heat transfer, which is presented in Fig. 12.

### Streamlines profiles

**Figure 13:**
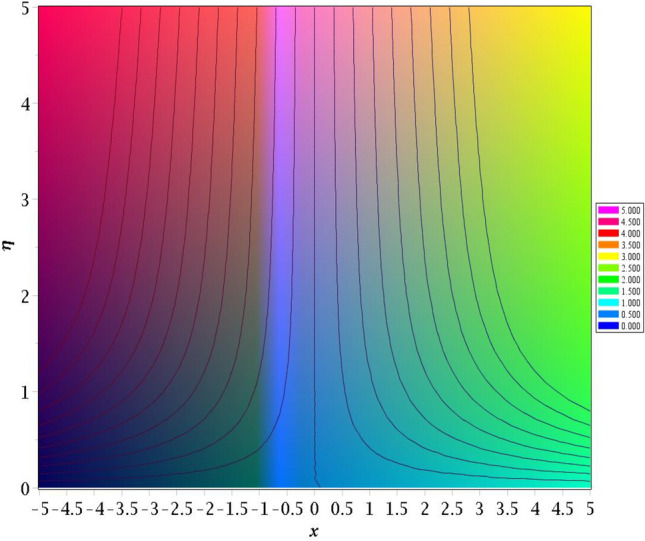
Stream lines for *M* = 0.2.

**Figure 14:**
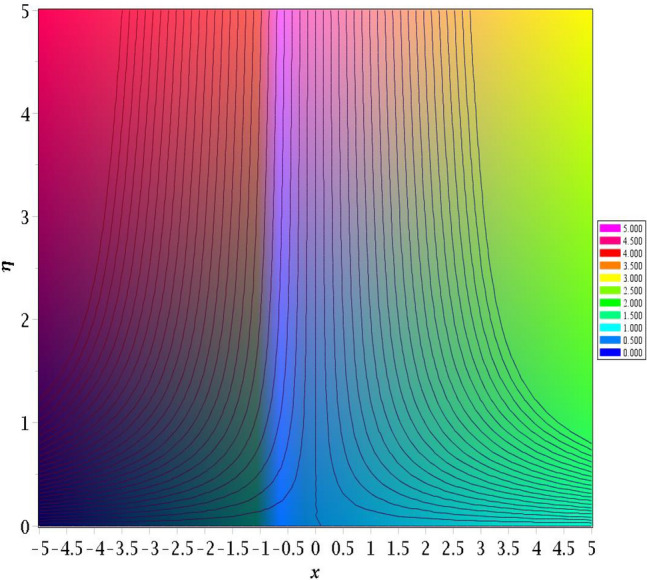
Stream lines for *M* = 0.4.

Streamlines, especially when applied to investigate fluid behaviour and the depiction of flow, offer a variety of characteristics that, when taken into consideration combined and enable them to be effective instruments for understanding and doing research on the motions of fluids. Figures 13 and 14 exhibit magnetic parameter for various values of *M* = 0.2, 0.4 influences on streamlines plots. Magnetic parameter strength draws electrical conductivity molecules more towards to the main stream.

## Conclusion

A numerical study has been conducted on the magnetized hybrid nanofluid flow over the stretching cylinder with porosity effect. The mathematical system’s partial differential equations are converted into ordinary differential equations by using the similarity transformations and the results are numerically sketched by using the BVP Midrich technique in MAPLE solver.

The crucial findings are:

- Increasing porosity and magnetic field inputs result in diminishing behavior of the SWCNT’s/TiO_2_ and MWCNT’s/TiO_2_ velocity profiles.
- The velocity profile enhanced with the higher values of curvature parameter.
- Additional properties of magnetic and Eckert number parameter inputs result in improved temperature profiles.
- The energy profile decreased with increasing the values of the Prandtl and curvature parameters.
- The energy profile enhanced by about 62% when the radiation parameter values increases from 1.0 to 3.0.
- The skin friction profile decreased behaviour for growing estimation of the magnetic field parameter (M) due to Lorentz force.
- In this hybrid model, the Nusselt number profile increased tendency for the higher values of the magnetic field parameter.

## Data Availability

The datasets used and/or analysed during the current study available from the corresponding author on reasonable request.

## List of symbols

*a*: Radius of the cylinder
*B*: Magnitude of the magnetic field
*C*_*f*_: Skin friction coefficient
*C*_*p*_: Specific heat at constant temperature (J kg^−1^ K^−1^)
*Ec*: Eckert number
*k*: Thermal conductivity (W m^−1^ K^−1^)
*L*: Characteristic length of the sheet
*M*: Magnetic parameter
*Nu*_*x*_: Local Nusselt number
Pr: Prandtl number
*q*_*w*_: Surface heat flux
Re_*x*_: Local Reynolds number
*x*, *r*: Cylinder coordinates (unit: m)
*S*: Mass transpiration parameter
*t*: Time
*T*: Fluid temperature (K)
*T*_0_: Characteristic temperature (constant)
*v*_*w*_: Velocity of the wall mass transfer
*MHD*: Magnetohydrodynamics
*Pr*: Prandtl number
*Rd*: Thermal radiation
*T*_*w*_: Wall temperature (K)
*T*_∞_: Ambient temperature (K)
*u*, *v*: Fluid velocities in the *x*; r-directions, respectively (unit: m s^−1^)
*u*_0_: Characteristic velocity (constant)
*U*_*w*_: Reference velocity of the stretching/shrinking cylinder (unit: m s^−1^)

## Greek symbols

$\phi_{1} ,\phi_{2}$: Nanoparticles volume fractions
*η*: Similarity variable
*γ*: Curvature parameter
*λ*: Stretching/shrinking parameter
*μ*: Dynamic viscosity (kg m^−1^ s^−1^)
$$\upsilon$$: Kinematic viscosity (m^2^ s^−1^)
*θ*: Dimensionless fluid temperature
*ρ*: Fluid density (kg m^−3^)
*τ*: Dimensionless time variable
*τ*_*w*_: Wall shear stress
*σ*: Electrical conductivity (S m^−1^)

## Subscripts

*f*, *nf*, *hnf*: Base fluid, nanofluid and hybrid nanofluid
*s*_1_, *s*_2_: First and second solid nanoparticles
_′_: Differentiation with respect to *η*
